# Mitophagy‐Oxidative Stress Molecular Subtypes Define an Immunosuppressive Ecosystem and Vulnerabilities in Glioblastoma

**DOI:** 10.1111/jcmm.71154

**Published:** 2026-05-16

**Authors:** Changjiang He, Yang Wang, Hongliang Zhong

**Affiliations:** ^1^ Beijing Chaoyang Hospital, The Third Clinical Medical College Capital Medical University Beijing China; ^2^ Department of Neurosurgery, Beijing Chaoyang Hospital Capital Medical University Beijing China

**Keywords:** glioblastoma, immunotherapy, mitophagy oxidative stress, prognostic signature, tumour microenvironment

## Abstract

Glioblastoma (GBM) is an aggressive brain tumour with an immunosuppressive environment and poor prognosis; however, the roles of mitophagy and oxidative stress in its prognosis remain underexplored. This study analysed multi‐omics data from TCGA‐GBM (*n* = 168) and two GEO cohorts (GSE43378, *n* = 50; GSE147352, *n* = 85), compiling 603 mitophagy and oxidative stress‐related genes. Unsupervised consensus clustering identified molecular subtypes, and prognostic genes were found via univariate Cox regression, leading to a refined gene signature using LASSO. The tumour immune microenvironment was characterized with ESTIMATE, CIBERSORT, and ssGSEA, while immunotherapy response was predicted using TIDE and IPS. Drug sensitivity was evaluated with GDSC and CTRP data, and a clinical nomogram integrating the gene signature and clinical variables was constructed and validated. Two molecular subtypes, C1 and C2, showed different prognoses (HR = 0.64, *p* = 0.011) and immune profiles. A 7‐gene signature indicated high‐risk patients had worse survival (*p* < 0.001) and an immunosuppressive environment. The risk score correlated with anti‐PD‐1 response (*p* < 0.05) and predicted therapy sensitivity. The signature was an independent prognostic factor (HR = 3.37, *p* < 0.001), and the nomogram accurately predicted survival at 1, 3, and 5 years. An innovative prognostic signature from mitophagy and oxidative stress genes stratifies GBM patients, characterizes the immunosuppressive microenvironment, and identifies therapeutic vulnerabilities for personalized treatment.

## 1 Introduction

Glioblastoma (GBM), designated as a Grade IV glioma by the World Health Organization, constitutes the most prevalent and fatal primary malignant brain tumour in adults [1]. The existing standard treatment protocol, which includes maximal safe surgical resection followed by concurrent radiochemotherapy with temozolomide, provides only a limited survival advantage, with a median overall survival of approximately 12–15 months and a 5‐year survival rate of less than 10% [2]. The significant inter‐ and intra‐tumoral heterogeneity, along with a markedly immunosuppressive tumour microenvironment (TME), are major factors contributing to therapeutic resistance and tumour recurrence [3, 4]. Consequently, elucidating the molecular mechanisms underlying GBM aggressiveness and identifying reliable prognostic biomarkers are essential for the development of novel therapeutic strategies.

In addition to genetic alterations, dysregulated cellular processes are fundamental characteristics of cancer that contribute to tumour progression and resistance to treatment [5]. Mitophagy, which refers to the selective autophagy of damaged mitochondria, and oxidative stress, characterized by an imbalance between the production and elimination of reactive oxygen species (ROS), are two intrinsically interconnected processes essential for the maintenance of cellular homeostasis [6]. Their roles in cancer are multifaceted and dependent on specific contexts. Moderate oxidative stress levels can facilitate pro‐tumorigenic signalling, enhance proliferation, and enable adaptation to hypoxic conditions, whereas excessive oxidative stress can lead to cell death [7]. Similarly, mitophagy can inhibit tumorigenesis by preserving genomic stability or alternatively support cancer cell survival by recycling nutrients and facilitating metabolic reprogramming under stress conditions [8, 9].

In glioblastoma multiforme (GBM), hypoxic niches and metabolic alterations are prominent characteristics, indicating that mitophagy and oxidative stress pathways are likely crucial for tumour maintenance and progression [10, 11]. Prior research has frequently concentrated on individual genes or pathways, thereby lacking a holistic perspective on the collective prognostic and immunological implications of mitophagy and oxidative stress‐related genes (MORGs) in GBM. A systems‐level analysis that integrates these processes could offer novel insights into the biology of GBM and identify new therapeutic targets.

To address this research gap, we conducted an integrative multi‐omics analysis of glioblastoma (GBM) cohorts sourced from public databases. We posited that mitophagy and oxidative stress‐related genes (MORGs) delineate molecular subtypes with distinct clinical outcomes and immune landscapes, and that a gene expression signature derived from these genes could function as a robust prognostic biomarker. The objectives of our study are to: (1) molecularly stratify GBM patients based on MORG expression profiles; (2) develop and validate a prognostic gene signature; (3) characterize the associated immune microenvironment and response to immunotherapy; and (4) identify potential therapeutic vulnerabilities. This research provides a valuable resource for elucidating the roles of mitophagy and oxidative stress in GBM, and offers a clinically translatable tool for prognosis prediction and treatment guidance.

## 2 Materials and Methods

### 2.1 Data Acquisition and Processing

Publicly available transcriptomic and corresponding clinical data for glioblastoma (GBM) were retrieved from The Cancer Genome Atlas (TCGA) database via the TCGAbiolinks package (test set: TCGA‐GBM, *n* = 168) and the Gene Expression Omnibus (GEO) database using GEOquery (validation sets: GSE43378, *n* = 50; GSE147352, *n* = 85) [12, 13, 14]. Somatic mutation and copy number variation (CNV) data were obtained from cBioPortal and processed using GISTIC2.0 [15, 16]. A compendium of 603 mitophagy and oxidative stress‐related genes (MORGs) was curated by intersecting genes from the GeneCards database (keywords: ‘Mitophagy’, ‘Oxidative stress’; filter: Protein Coding, Score > 2) and relevant literature with the expression matrices of the aforementioned datasets [17, 18, 19].

### 2.2 Compilation of Mitophagy and Oxidative Stress‐Related Genes

Genes associated with mitophagy and oxidative stress were systematically curated. The GeneCards database [20] was queried using the keywords ‘Mitophagy’ and ‘Oxidative stress’. Protein‐coding genes with a relevance score > 2 were selected, yielding 1633 mitophagy‐related genes (MRGs) and 2899 oxidative stress‐related genes (ORGs). These lists were supplemented with published gene sets from relevant literature [21, 22]. After merging and removing duplicates, the intersection of MRGs and ORGs with genes expressed in all three analysed datasets (TCGA‐GBM, GSE43378, GSE147352) defined our final set of 603 mitophagy and oxidative stress‐related genes (MORGs) (Table 1).

**TABLE 1:** Patient characteristics of GBM patients in the TCGA datasets.

Characteristics	Overall(*n* = 168)
Age, median (IQR)	60 (50.75, 69)
Gender, *n* (%)	
Male	109 (64.9%)
Female	59 (35.1%)
OS, *n* (%)	
1	136 (81%)
0	32 (19%)
OS.time, median (IQR)	360 (159.25, 532.75)

Abbreviations: GBM, glioblastoma multiforme; TCGA, The Cancer Genome Atlas.

### 2.3 Analysis of Genomic Alterations

The somatic mutation landscape of the 603 MORGs in TCGA‐GBM samples was analysed and visualized using the maftools package [23]. To identify significant recurrent copy number alterations, GISTIC 2.0 analysis [24] was performed on the ‘Masked Copy Number Segment’ data from TCGA‐GBM. Focal amplifications and deletions were identified with a confidence level of 0.95.

### 2.4 Molecular Subtyping via Consensus Clustering

To identify prognostic molecular subtypes, univariate Cox regression was first performed on the expression of MORGs in the TCGA cohort to select genes significantly associated with overall survival (OS) (*p* < 0.05), defined as Prognostic MORGs (PMORGs). Consensus clustering was then applied to the expression matrix of these PMORGs using the ConsensusClusterPlus package [25]. Parameters were set as follows: maximum cluster number (*k*) = 10, 80% item resampling for 100 iterations, cluster algorithm = ‘km’, distance = ‘euclidean’. The optimal number of clusters was determined by consensus cumulative distribution function (CDF) and delta area plots. Survival differences between the resulting subtypes were assessed using Kaplan–Meier analysis.

### 2.5 Functional Enrichment and Pathway Analysis

Gene Set Variation Analysis (GSVA) [26] was conducted using the hallmark gene sets (h.all.v7.4.symbols.gmt) from the Molecular Signatures Database (MSigDB) [22, 27] to evaluate pathway activity differences between the identified subtypes. Differential expression analysis between subtypes was performed with the limma package [28] (|log_2_FC| > 0.5, adjusted *p*‐value < 0.05). The intersection of differentially expressed genes (DEGs) and PMORGs yielded Prognostic Mitophagy and Oxidative stress‐Related Differentially Expressed Genes (PMORDEGs). Functional annotation of PMORDEGs was performed via Gene Ontology (GO) [29] and Kyoto Encyclopedia of Genes and Genomes (KEGG) [30] pathway enrichment analyses using clusterProfiler [31]. Gene Set Enrichment Analysis (GSEA) [32] was further applied to all genes ranked by log_2_FC to identify subtle but coordinated pathway changes (Table 2).

**TABLE 2:** Results of GSVA.

ID	log FC	Ave Expr	*t*	*p*	adj. *p* val	*B*
HALLMARK_E2F_TARGETS	0.20796	0.003312	3.429284	0.000747	0.001205	1.77271
HALLMARK_MYC_TARGETS_V1	0.183698	0.00981	3.31106	0.001119	0.001697	2.14875
HALLMARK_G2M_CHECKPOINT	0.169034	0.00323	2.895524	0.004244	0.005895	3.37645
HALLMARK_MYC_TARGETS_V2	0.156076	0.00989	2.835745	0.005085	0.006519	3.54079
HALLMARK_SPERMATOGENESIS	0.136998	0.01494	3.930412	0.00012	0.0002	0.0521
HALLMARK_PANCREAS_BETA_CELLS	0.123538	0.008997	2.866207	0.004639	0.006269	3.45744
HALLMARK_PEROXISOME	0.07819	0.00972	2.05262	0.041527	0.049437	5.3973
HALLMARK_NOTCH_SIGNALLING	0.09754	0.004412	2.13828	0.033815	0.041238	5.22161
HALLMARK_ADIPOGENESIS	0.10132	0.00871	2.51691	0.012694	0.015868	4.36379
HALLMARK_MTORC1_SIGNALLING	0.12301	0.01012	2.8406	0.005011	0.006519	3.52756
HALLMARK_COMPLEMENT	0.37188	0.00853	9.85929	1.13 e‐18	7.06 e‐18	31.58752
HALLMARK_IL2_STAT5_SIGNALLING	0.37643	0.00456	10.7532	3.13 e‐21	3.91 e‐20	37.42665
HALLMARK_ALLOGRAFT_REJECTION	0.37747	0.004211	7.80129	4.46 e‐13	1.72 e‐12	18.84761
HALLMARK_INTERFERON_GAMMA_RESPONSE	0.38165	0.004576	7.3592	5.98 e‐12	1.99 e‐11	16.29171
HALLMARK_COAGULATION	0.40454	0.003259	11.3568	5.52 e‐23	1.38 e‐21	41.43409
HALLMARK_ANGIOGENESIS	0.40958	0.004269	8.76677	1.22 e‐15	5.55 e‐15	24.67408
HALLMARK_INFLAMMATORY_RESPONSE	0.4393	0.00136	9.93513	6.89 e‐19	4.92 e‐18	32.07744
HALLMARK_EPITHELIAL_MESENCHYMAL_TRANSITION	0.44654	0.01811	10.0813	2.65 e‐19	2.21 e‐18	33.02455
HALLMARK_IL6_JAK_STAT3_SIGNALLING	0.49652	0.002686	11.0456	4.45 e‐22	7.41 e‐21	39.36226
HALLMARK_TNFA_SIGNALLING_VIA_NFKB	0.50089	0.00812	12.1582	2.46 e‐25	1.23 e‐23	46.80965

Abbreviations: GSVA, Gene Set Variation Analysis; TCGA, The Cancer Genome Atlas.

### 2.6 Characterization of the Tumour Immune Microenvironment

The immune landscape was profiled using multiple complementary approaches. The ESTIMATE algorithm [33] was used to compute stromal, immune, and ESTIMATE scores for each sample. The infiltration abundances of 28 immune cell types were quantified via single‐sample Gene Set Enrichment Analysis (ssGSEA) based on well‐curated gene signatures [34]. The expression profiles of 47 known immune checkpoint genes [35] and 27 HLA family genes were extracted and compared across subtypes. To infer potential response to immunotherapy, immunophenoscore (IPS) data for GBM were downloaded from The Cancer Immunome Atlas (TCIA) and analyzed.

### 2.7 Construction of a Prognostic Gene Signature Using LASSO Regression

To develop a robust prognostic model, Least Absolute Shrinkage and Selection Operator (LASSO) Cox regression [36, 37] was applied to the PMORDEGs in the TCGA cohort to prevent overfitting and select the most informative genes. Ten‐fold cross‐validation was used to determine the optimal penalty parameter (*λ*). A risk score for each patient was calculated using the formula: RiskScore = Σ (Coefficient_i * Expression_i). Patients were stratified into high‐ and low‐risk groups based on the median risk score. The prognostic performance of the signature was evaluated by Kaplan–Meier survival analysis and time‐dependent receiver operating characteristic (ROC) curve analysis (1‐, 3‐, 5‐year). Validation of the risk stratification was performed in the two independent GEO datasets.

### 2.8 Construction of Regulatory Networks

To explore the post‐transcriptional and transcriptional regulation of the key prognostic genes identified by LASSO, three interaction networks were constructed:

Protein–Protein Interaction (PPI) Network: The physical and functional interactions among key gene‐encoded proteins were retrieved from the STRING database [38] (minimum required interaction score: 0.015) and visualized using Cytoscape [39].

miRNA‐mRNA Regulatory Network: Potential miRNAs targeting the key genes were predicted using the ENCORI database (starBase v3.0) [40]. Interaction pairs supported by CLIP‐seq data in more than 8 cancer types (pancancerNum > 8) were retained to construct the network.

Transcription Factor (TF)‐mRNA Regulatory Network: TFs potentially binding to the promoters of the key genes were identified by mining ChIP‐seq data from the CHIPBase database (v3.0) [40]. TF‐gene pairs with the sum of supporting upstream and downstream samples > 5 were selected for network construction.

### 2.9 Analysis of Immunotherapy Response and Drug Sensitivity

The translational value of the prognostic signature was further assessed. The risk score was applied to a published melanoma immunotherapy cohort (Hugo et al. [41]) with available response data to compare scores between responders (R) and non‐responders (NR). To identify potential therapeutic agents, the correlation between the expression of the key genes and the sensitivity (IC50) to hundreds of compounds was analysed using data from the Genomics of Drug Sensitivity in Cancer (GDSC) [42] and Cancer Therapeutics Response Portal (CTRP) databases, integrated via the GSCA online platform [43].

### 2.10 Development and Assessment of a Clinical Prognostic Nomogram

To facilitate clinical translation, a multivariate Cox proportional hazards model was built by integrating the gene signature risk score with key clinical parameters (age, gender). This model was presented as a quantitative nomogram to predict 1‐, 2‐, and 3‐year survival probabilities. The predictive accuracy and discriminative ability of the nomogram were evaluated using calibration plots [44] and decision curve analysis (DCA) [45, 46], which quantifies the clinical net benefit across different threshold probabilities.

### 2.11 Statistical Analysis

All bioinformatics and statistical analyses were conducted using R software (version 4.2.2). Inter‐group differences for continuous variables were compared using Student's *t*‐test (normally distributed) or the Mann–Whitney U test (non‐normally distributed). Categorical variables were compared using the Chi‐square test. Survival differences were assessed with the log‐rank test. Correlations were analysed using Spearman's rank correlation coefficient. A two‐sided *p*‐value < 0.05 was considered statistically significant.

## 3 Results

### 3.1 Technology Roadmap

**FIGURE 1:**
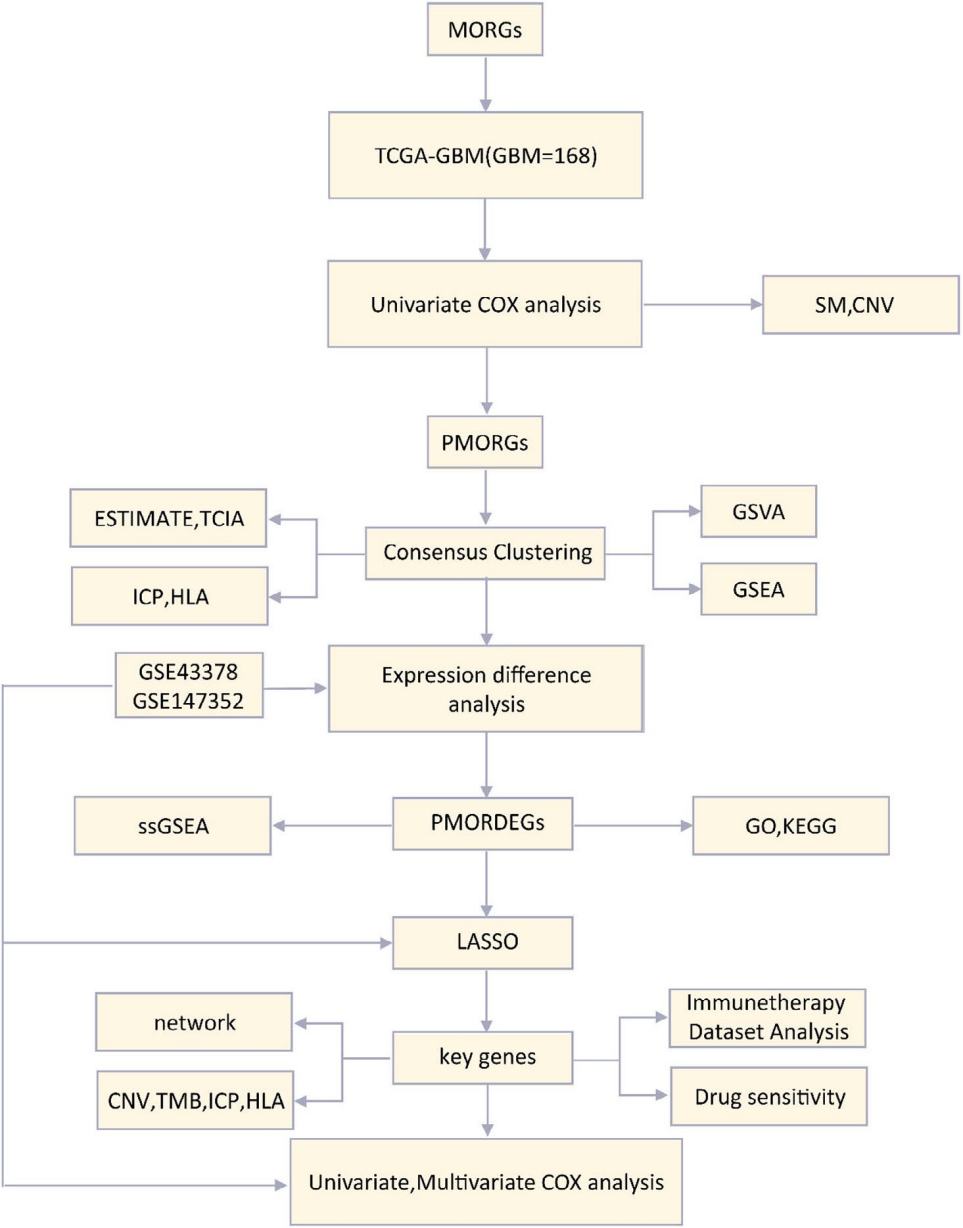
Technology roadmap.

MORGs, Mitophagy and Oxidative stress related genes; PMORGs, Prognostic Mitophagy and Oxidative stress related genes; SM, Somatic Mutation; SNV, Single Nucleotide Variant; CNV, Copy Number Variations; PMORDEGs, Prognostic Mitophagy and Oxidative stress related differentially expressed genes; ssGSEA, single‐sample gene‐set enrichment analysis; TCGA, The cancer genome atlas; GBM, Glioblastoma multiforme; ICP, Immune checkpoint; GSEA, Gene Set Enrichment Analysis; GSVA, Gene Set Variation Analysis; GO, Gene Ontology; KEGG, Kyoto Encyclopedia of Genes and Genomes; LASSO, Least absolute shrinkage and selection operator (Figure 1).

### 3.2 Genomic Alterations in Mitophagy and Oxidative Stress‐Related Genes

**FIGURE 2:**
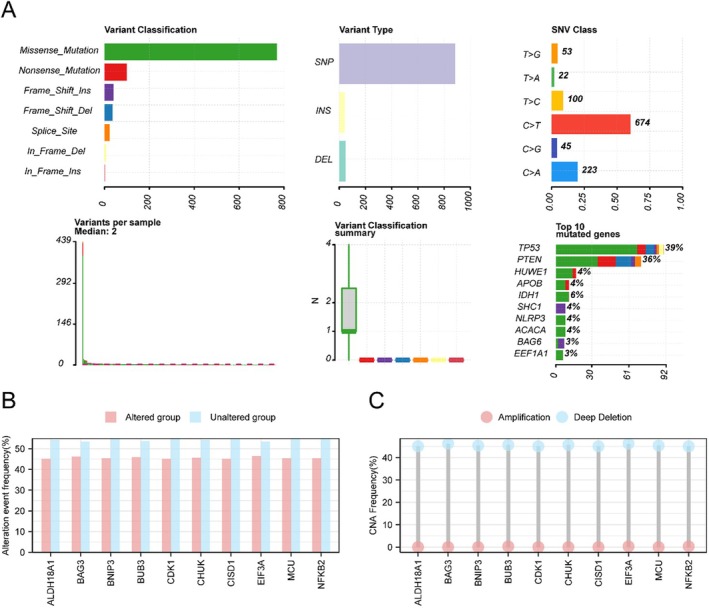
Genomic alterations in mitophagy and oxidative stress‐related genes (MORGs) in GBM. (A) Summary of somatic mutations in 603 MORGs from TCGA‐GBM patients. Top 10 mutated genes are shown. (B) Frequency of copy number variations (CNVs) in the top 10 MORGs. (C) Distribution of CNV types (amplification vs. deletion) for the top 10 MORGs.

We first analysed somatic mutations (SMs) and copy number variations (CNVs) of 603 mitophagy and oxidative stress‐related genes (MORGs) in GBM patients from the TCGA‐GBM cohort. Somatic mutation analysis revealed that missense mutations were the predominant type, with single nucleotide polymorphisms (SNPs) representing the majority of alterations (Figure 2A). The most frequent SNV was C‐to‐T substitution. Among the top 10 mutated genes, TP53 (39%) and PTEN (36%) exhibited the highest mutation rates.

Copy number analysis identified focal amplifications and deletions in 10 genes (ALDH18A1, BAG3, BNIP3, BUB3, CDK1, CHUK, CISD1, EIF3A, MCU, NFKB2), with an overall CNV frequency of approximately 45% (Figure 2B). Deletions were more prevalent than amplifications within the variant group (Figure 2C).

### 3.3 Identification of GBM Subtypes Based on Prognostic MORGs

**FIGURE 3:**
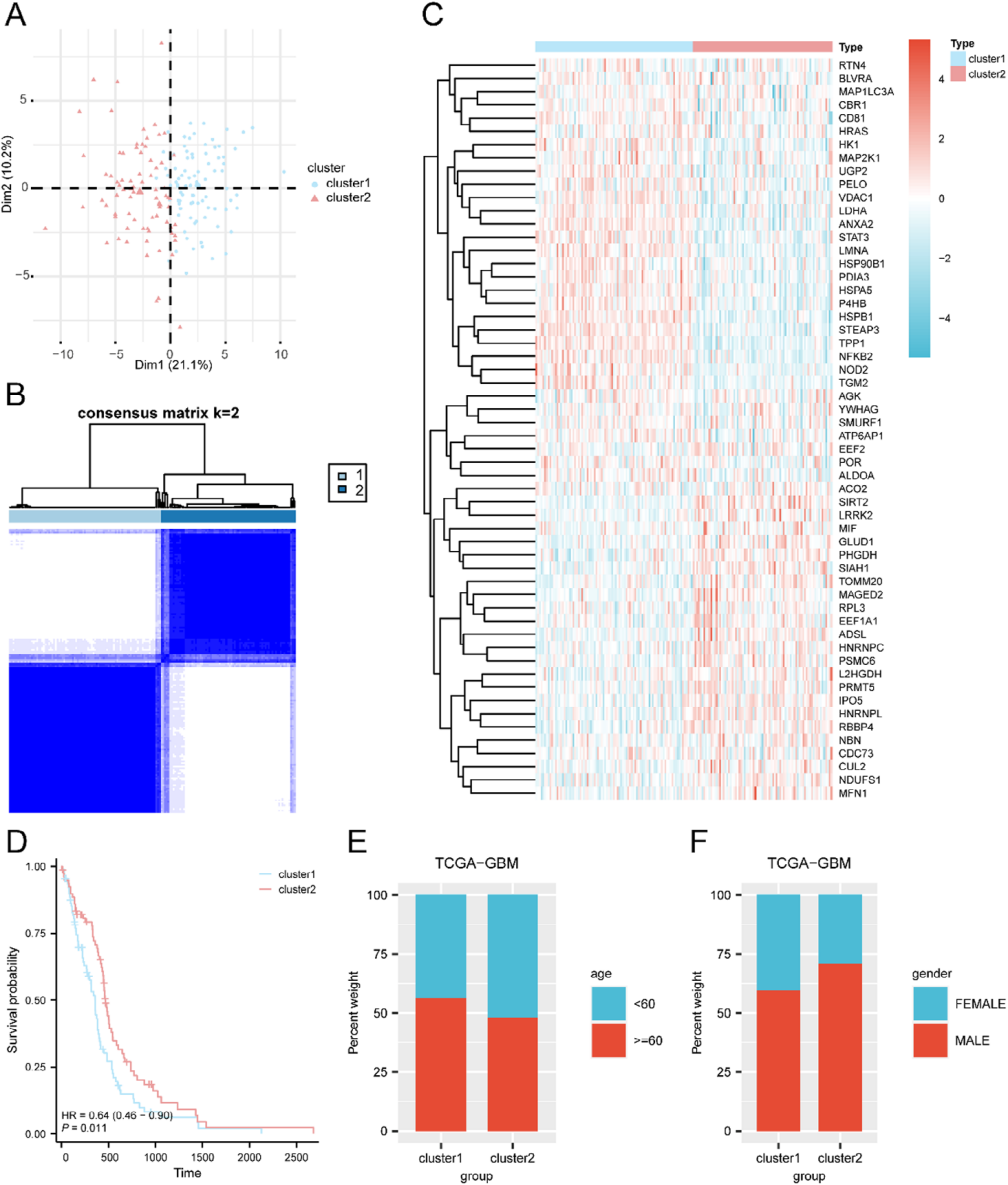
Molecular subtyping of GBM based on prognostic MORGs. (A) Principal component analysis (PCA) plot showing separation between two GBM subtypes (Subtype A and B). (B) Consensus clustering matrix. (C) Heatmap of prognostic MORG expression across subtypes. (D) Kaplan–Meier survival curves for the two subtypes. (E, F) Distribution of age (E) and gender (F) across subtypes.

Univariate Cox regression identified 56 prognostic MORGs (PMORGs, *p* < 0.05). Consensus clustering based on these PMORGs classified GBM patients into two distinct molecular subtypes: Subtype C1 (*n* = 89) and Subtype C2 (*n* = 79). Principal component analysis (PCA) demonstrated clear separation between the subtypes (Figure 3A,B). Kaplan–Meier survival analysis revealed that Subtype B was associated with significantly better overall survival compared to Subtype A (HR = 0.64, 95% CI: 0.46–0.90, *p* = 0.011; Figure 3D). No significant differences in age or gender distribution were observed between subtypes (Figure 3E,F).

### 3.4 Pathway Enrichment and Differential Expression Analysis

**FIGURE 4:**
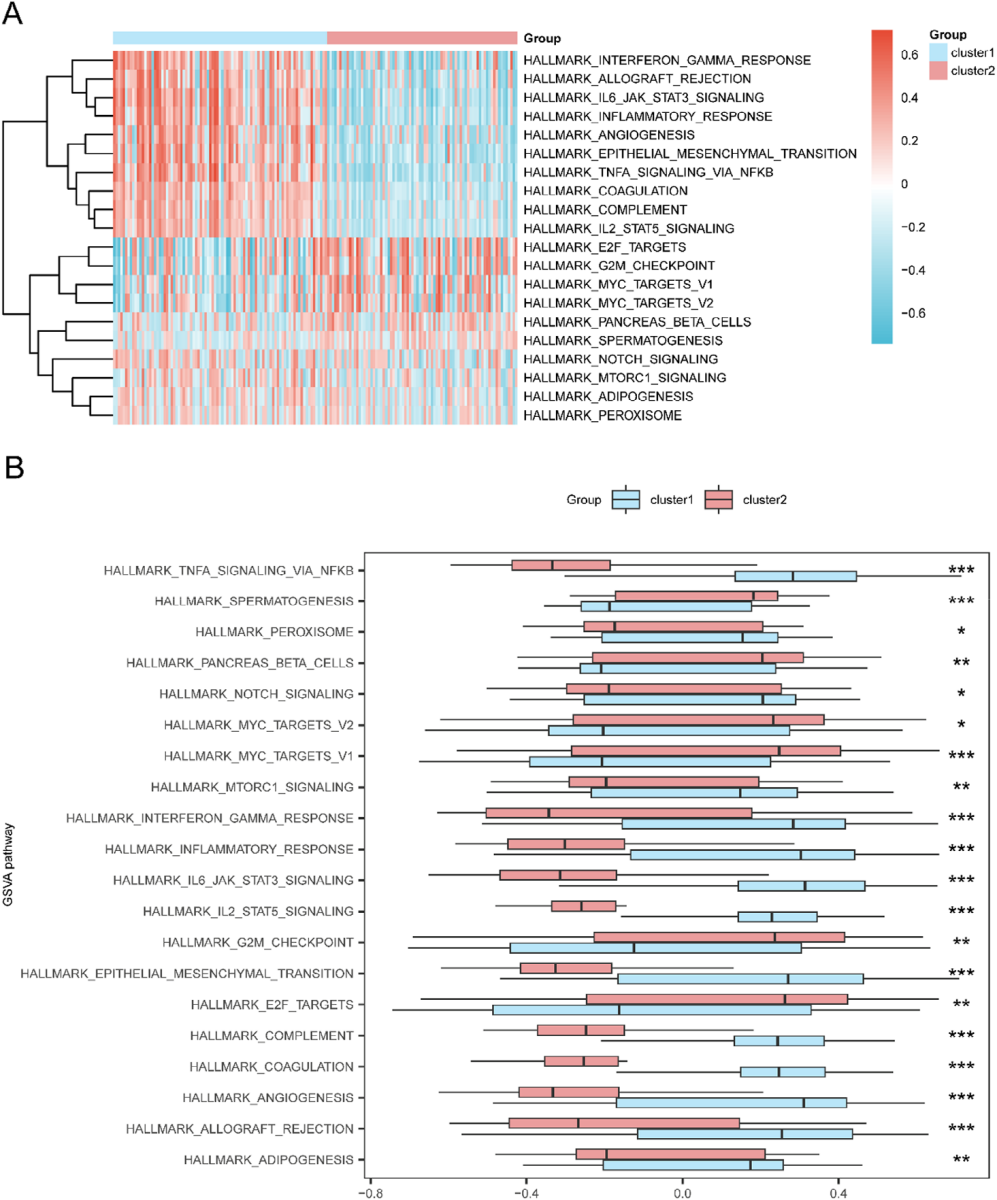
GSVA enrichment analysis of GBM subtypes. (A) Heatmap of GSVA scores for hallmark pathways differentially activated between Subtype A and B. (B) Box plots showing significant pathway activity differences. Pathways with |logFC| > 0.5 and adj. *p* < 0.05 are shown.

Gene Set Variation Analysis (GSVA) highlighted significant pathway activity differences between subtypes, including upregulation of E2F_TARGETS, MYC_TARGETS, G2M_CHECKPOINT, and MTORC1_SIGNALLING in Subtype A, while immune‐related pathways such as INFLAMMATORY_RESPONSE and TNFA_SIGNALLING_VIA_NFKB were enriched in Subtype B (Figure 4A,B).

**FIGURE 5:**
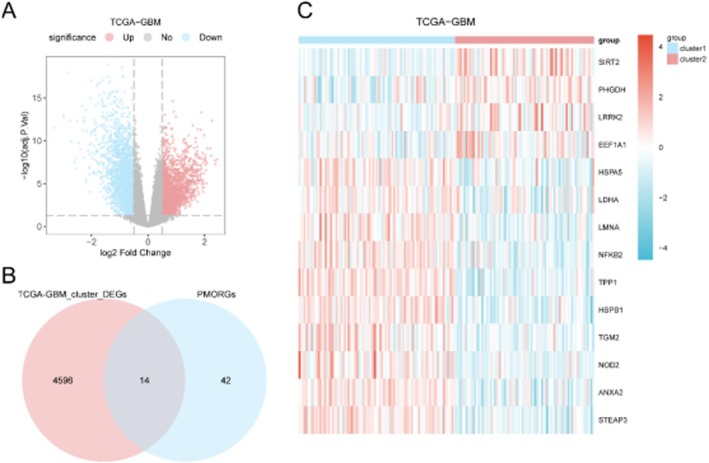
Differential gene expression between GBM subtypes. (A) Volcano plot of differentially expressed genes (DEGs) between Subtype A and B. (B) Venn diagram showing overlap between DEGs and prognostic MORGs (PMORGs). (C) Heatmap of 14 prognostic mitophagy and oxidative stress‐related differentially expressed genes (PMORDEGs) across subtypes.

Differential expression analysis identified 4610 genes significantly dysregulated between subtypes (|\text{logFC}| > 0.5, adj. *p* < 0.05; Figure 5A). Intersection with PMORGs yielded 14 prognostic mitophagy and oxidative stress‐related differentially expressed genes (PMORDEGs: TPP1, NFKB2, ANXA2, STEAP3, HSPB1, LMNA, NOD2, SIRT2, TGM2, HSPA5, LDHA, PHGDH, EEF1A1 and LRRK2; Figure 5B,C).

### 3.5 Functional Enrichment of PMORDEGs

**FIGURE 6:**
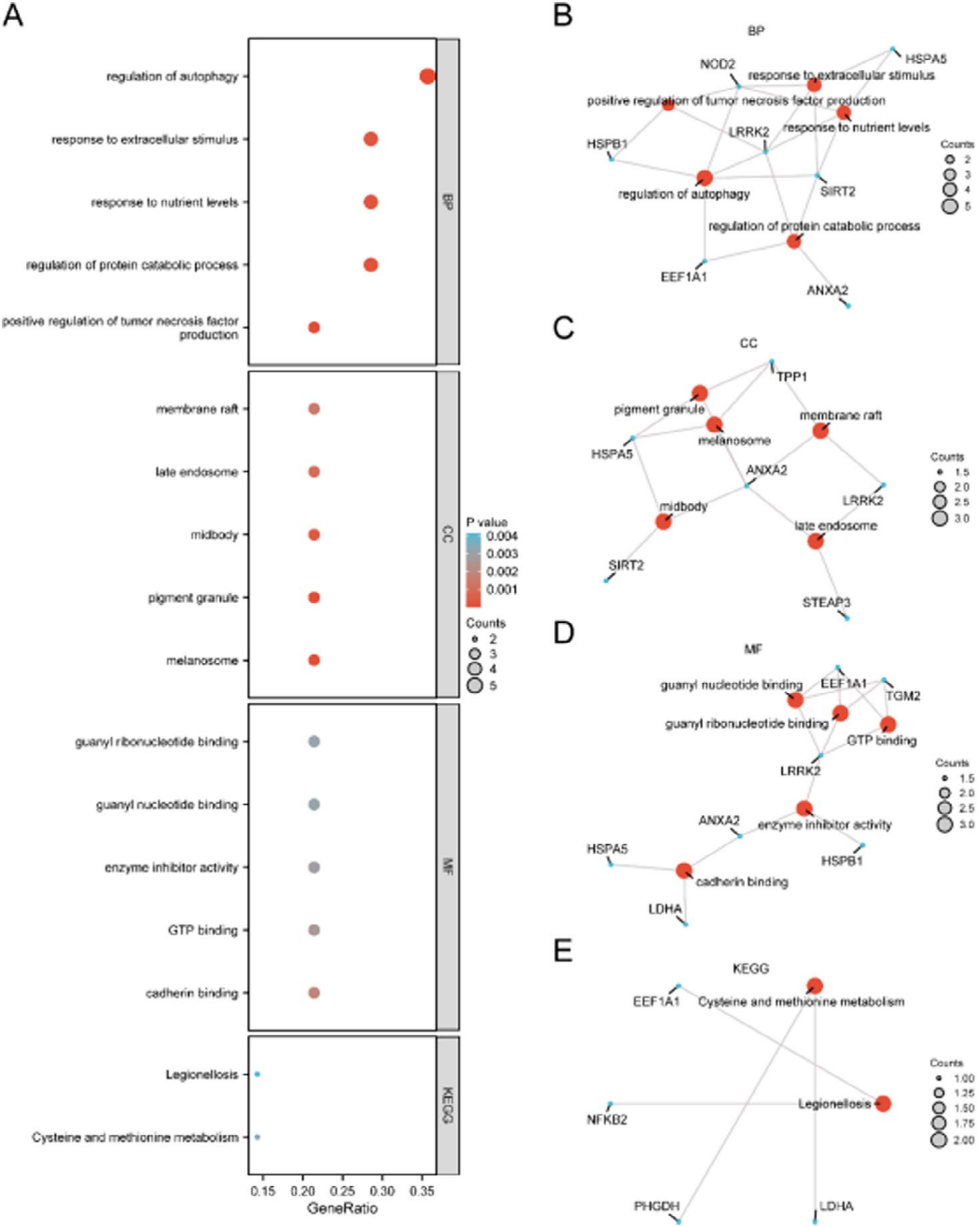
Functional enrichment analysis of PMORDEGs. (A) Bubble plot of significantly enriched Gene Ontology (GO) terms and KEGG pathways. (B–E) Network diagrams showing enriched biological processes (B), cellular components (C), molecular functions (D), and KEGG pathways (E). Blue nodes: Genes; red nodes: Pathways.

GO and KEGG enrichment analyses indicated that PMORDEGs were significantly involved in autophagy regulation, protein catabolism, response to nutrient levels, and immune‐related processes (Figure 6A–E). Key enriched terms included ‘regulation of autophagy’, ‘cadherin binding’, ‘GTP binding’, and pathways such as ‘Cysteine and methionine metabolism’ and ‘Legionellosis’ (Table 3).

**TABLE 3:** GO and KEGG enrichment analysis results of PMORDEGs.

Ontology	ID	Description	Gene ratio	Bg ratio	*p*	*p*.adjust	*q* value
BP	GO:0010506	Regulation of autophagy	5/14	336/18800	3.104 e‐06	0.00331816	0.0015814
BP	GO:0042176	Regulation of protein catabolic process	4/14	395/18800	0.00016257	0.0096551	0.00460151
BP	GO:0031667	Response to nutrient levels	4/14	446/18800	0.00025896	0.0138413	0.00659661
BP	GO:0009991	Response to extracellular stimulus	4/14	479/18800	0.00033999	0.01397868	0.00666209
BP	GO:0032760	Positive regulation of tumour necrosis factor production	3/14	103/18800	5.5639 e‐05	0.00819098	0.00390373
CC	GO:0042470	Melanosome	3/14	109/19594	5.8295 e‐05	0.00378916	0.00233179
CC	GO:0048770	Pigment granule	3/14	109/19594	5.8295 e‐05	0.00378916	0.00233179
CC	GO:0030496	Midbody	3/14	203/19594	0.00036662	0.01588671	0.00977644
CC	GO:0005770	Late endosome	3/14	283/19594	0.00096435	0.02507304	0.01542956
CC	GO:0045121	Membrane raft	3/14	326/19594	0.00144958	0.02715864	0.01671301
MF	GO:0045296	Cadherin binding	3/14	333/18410	0.00184092	0.04100642	0.0240919
MF	GO:0005525	GTP binding	3/14	379/18410	0.00266135	0.04100642	0.0240919
MF	GO:0004857	Enzyme inhibitor activity	3/14	390/18410	0.00288618	0.04100642	0.0240919
MF	GO:0019001	Guanyl nucleotide binding	3/14	401/18410	0.0031225	0.04100642	0.0240919
MF	GO:0032561	Guanyl ribonucleotide binding	3/14	401/18410	0.0031225	0.04100642	0.0240919
KEGG	hsa00270	Cysteine and methionine metabolism	2/14	51/8164	0.00331876	0.09498956	0.09346799
KEGG	hsa05134	Legionellosis	2/14	57/8164	0.00412998	0.09498956	0.09346799

Abbreviations: BP, biological process; CC, cellular component; GO, Gene Ontology; KEGG, Kyoto Encyclopedia of Genes and Genomes; MF, molecular function; PMORDEGs, Prognostic mitophagy and oxidative stress related differentially expressed genes.

**FIGURE 7:**
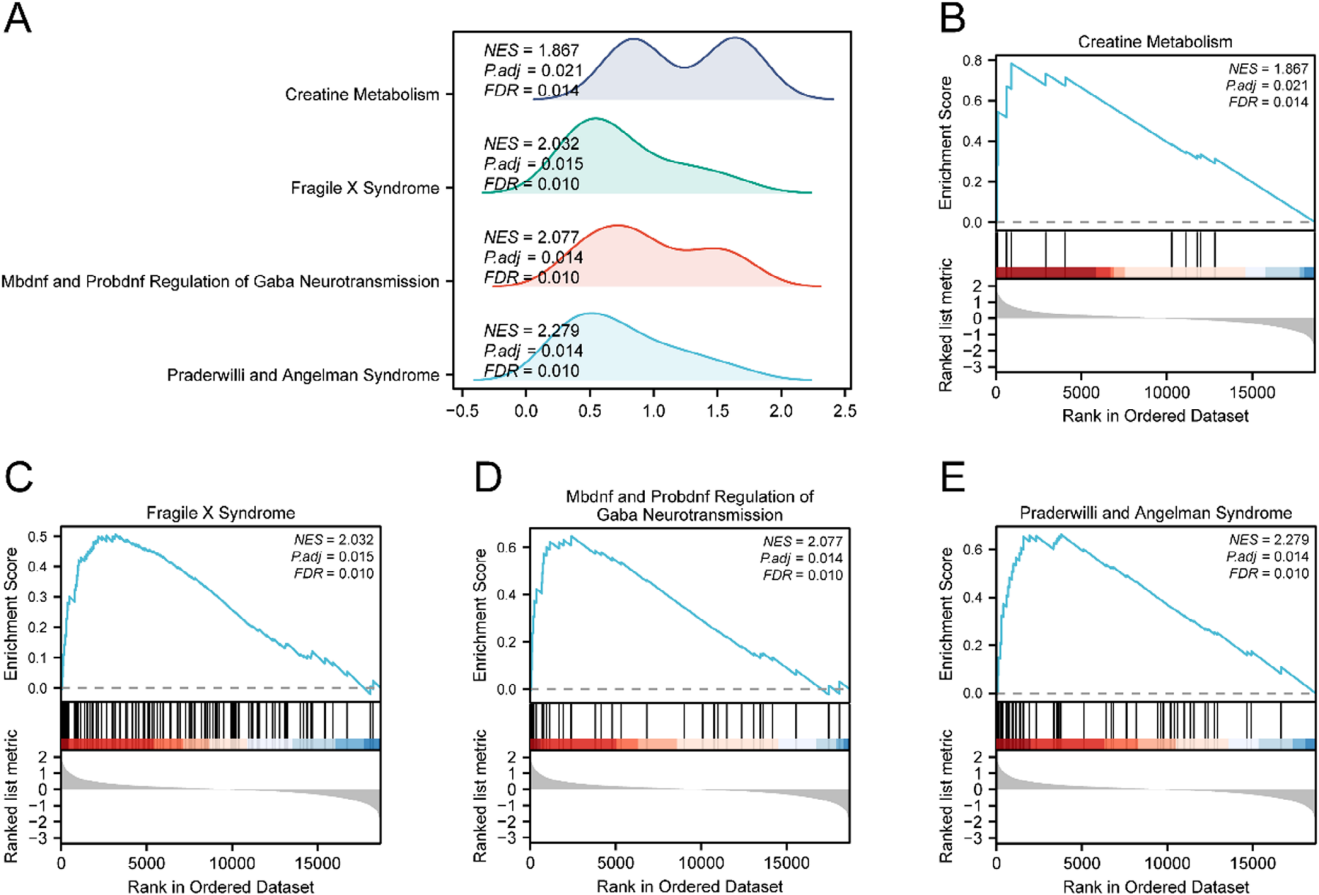
GSEA of GBM subtypes. (A) Mountain plot summarizing GSEA results. (B–E) Enrichment plots for significantly enriched pathways: CREATINE_METABOLISM (B) FRAGILE_X_SYNDROME (C) MBDNF_AND_PROBDNF_REGULATION_OF_GABA_NEUROTRANSMISSION (D) and PRADERWILLI_AND_ANGELMAN_SYNDROME (E).

GSEA further revealed that genes upregulated in Subtype B were enriched in pathways related to creatine metabolism, fragile X syndrome, GABA neurotransmission, and Prader–Willi/Angelman syndromes (Figure 7A–E, Table 4).

**TABLE 4:** GSEA enrichment analysis results of TCGA‐GBM dataset.

Description	setSize	enrichmentScore	NES	*p*	*p*.adjust	*q*
WP_PRADERWILLI_AND_ANGELMAN_SYNDROME	48	0.66270342	2.27885052	0.00235294	0.01442771	0.00966449
WP_MBDNF_AND_PROBDNF_REGULATION_OF_GABA_NEUROTRANSMISSION	35	0.64734976	2.0772346	0.00232019	0.01442771	0.00966449
WP_FRAGILE_X_SYNDROME	120	0.50578482	2.03245489	0.00266667	0.01537179	0.0102969
REACTOME_CREATINE_METABOLISM	11	0.78406068	1.86667139	0.00431034	0.0210943	0.01413015

Abbreviations: GBM, glioblastoma multiforme; GSEA, Gene Set Enrichment Analysis; TCGA, The Cancer Genome Atlas.

### 3.6 Immune Microenvironment Characterization

**FIGURE 8:**
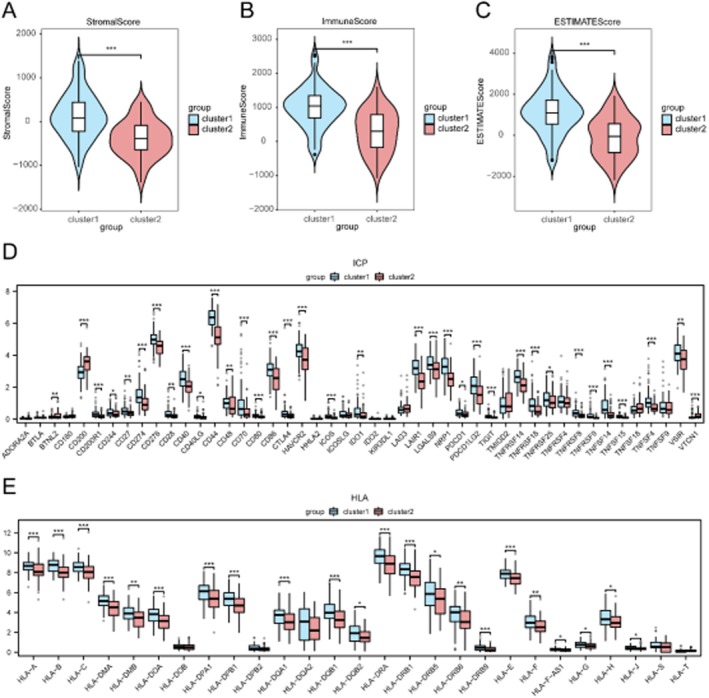
Immune microenvironment differences between GBM subtypes. (A–C) Comparison of stromal score (A), immune score (B), and ESTIMATE score (C) between subtypes. (D) Expression differences of 47 immune checkpoint genes. (E) Expression differences of 27 HLA family genes. *p < 0.05, **p < 0.01, ***p < 0.001.

**FIGURE 9:**
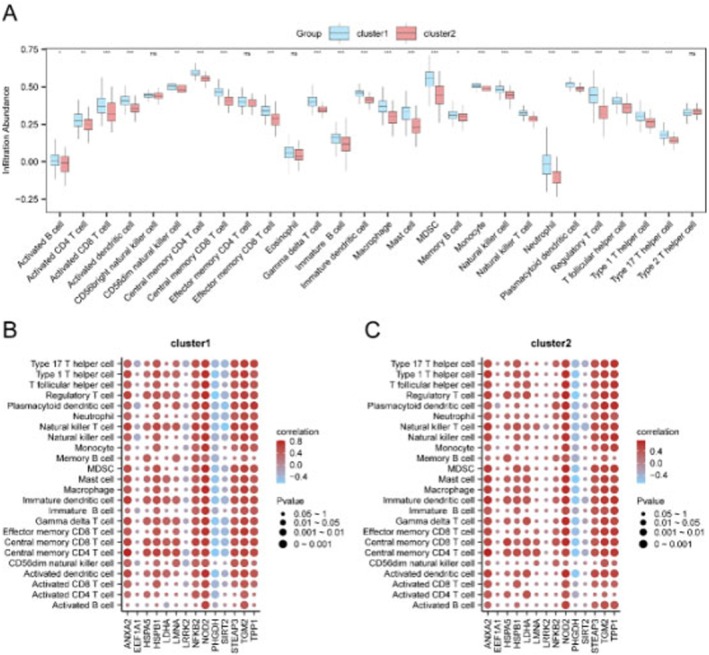
Immune cell infiltration analysis. (A) Comparison of immune cell infiltration levels between subtypes using ssGSEA. (B, C) Correlation heatmaps between PMORDEG expression and immune cell infiltration in Subtype A (B) and Subtype B (C).

Subtype B exhibited significantly higher stromal, immune and ESTIMATE scores compared to Subtype A (all *p* < 0.05; Figure 8A–C). Expression analysis revealed differential expression of 35 immune checkpoint genes and 22 HLA family genes between subtypes (Figure 8D,E). Immune cell infiltration analysis via ssGSEA showed significant differences in 24 immune cell types, with Subtype B demonstrating higher levels of activated CD4+ and CD8+ T cells, dendritic cells, and natural killer cells (Figure 9A). Correlation analysis indicated strong associations between PMORDEGs and immune cell abundances (Figure 9B,C).

### 3.7 Immunotherapy Response Prediction

**FIGURE 10:**
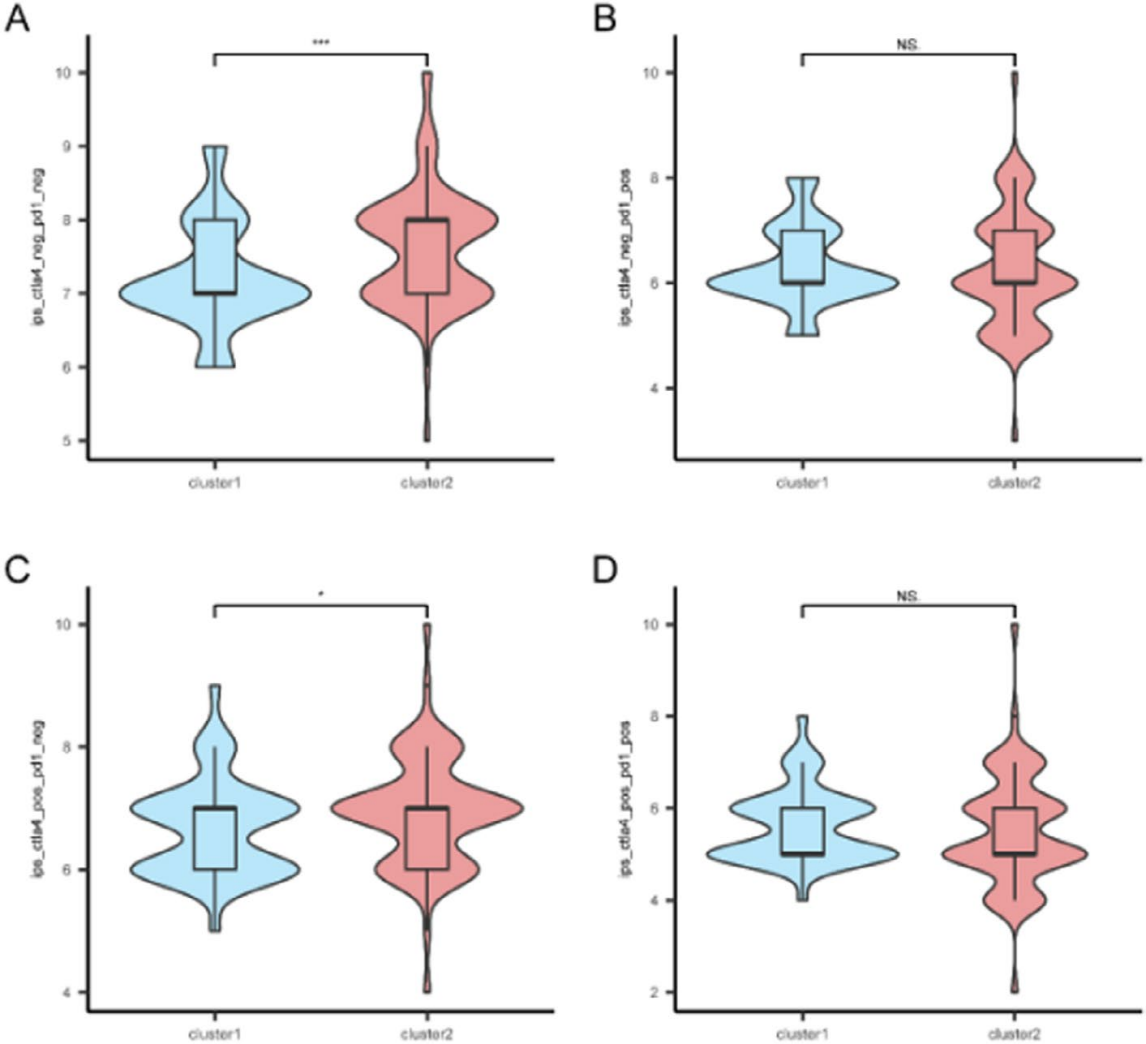
Immunotherapy response prediction. Box plots comparing immunophenoscore (IPS) (A), IPS‐PD1/PD‐L1/PD‐L2 (B), IPS‐CTLA4 (C), and IPS‐PD1/PD‐L1/PD‐L2 + CTLA4 (D) between GBM subtypes.

Analysis of immunophenoscore (IPS) data revealed that Subtype B had significantly higher IPS and IPS‐CTLA4 scores compared to Subtype A (*p* < 0.05), suggesting a potentially more favourable response to immune checkpoint inhibitors (Figure 10A,C).

### 3.8 Validation of Subtypes and PMORDEG Expression in Independent Cohorts

**FIGURE 11:**
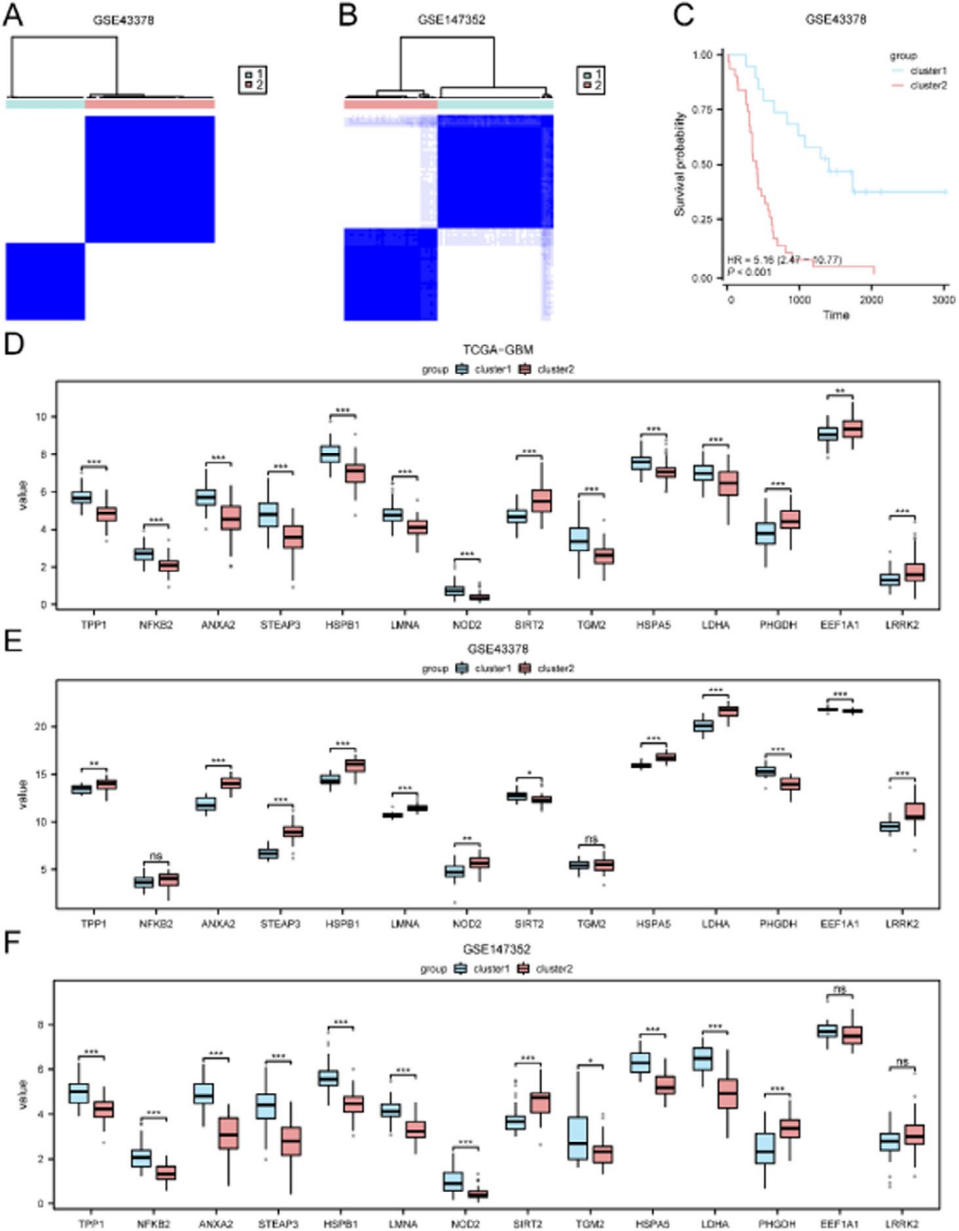
Validation of GBM subtypes and PMORDEG expression in independent datasets. (A, B) PCA plots of subtypes in GSE43378 (A) and GSE147352 (B). (C) Kaplan–Meier survival analysis in GSE43378. (D–F) Expression of PMORDEGs in TCGA (D), GSE43378 (E) and GSE147352 (F) datasets.

Consensus clustering in two independent GEO datasets (GSE43378 and GSE147352) confirmed the reproducibility of the two GBM subtypes (Figure 11A,B). Survival analysis in GSE43378 further validated the prognostic distinction between subtypes (*p* < 0.05; Figure 11C). Expression patterns of PMORDEGs were consistent across all three datasets (Figure 11D–F).

### 3.9 Construction and Validation of a Prognostic LASSO Model

**FIGURE 12:**
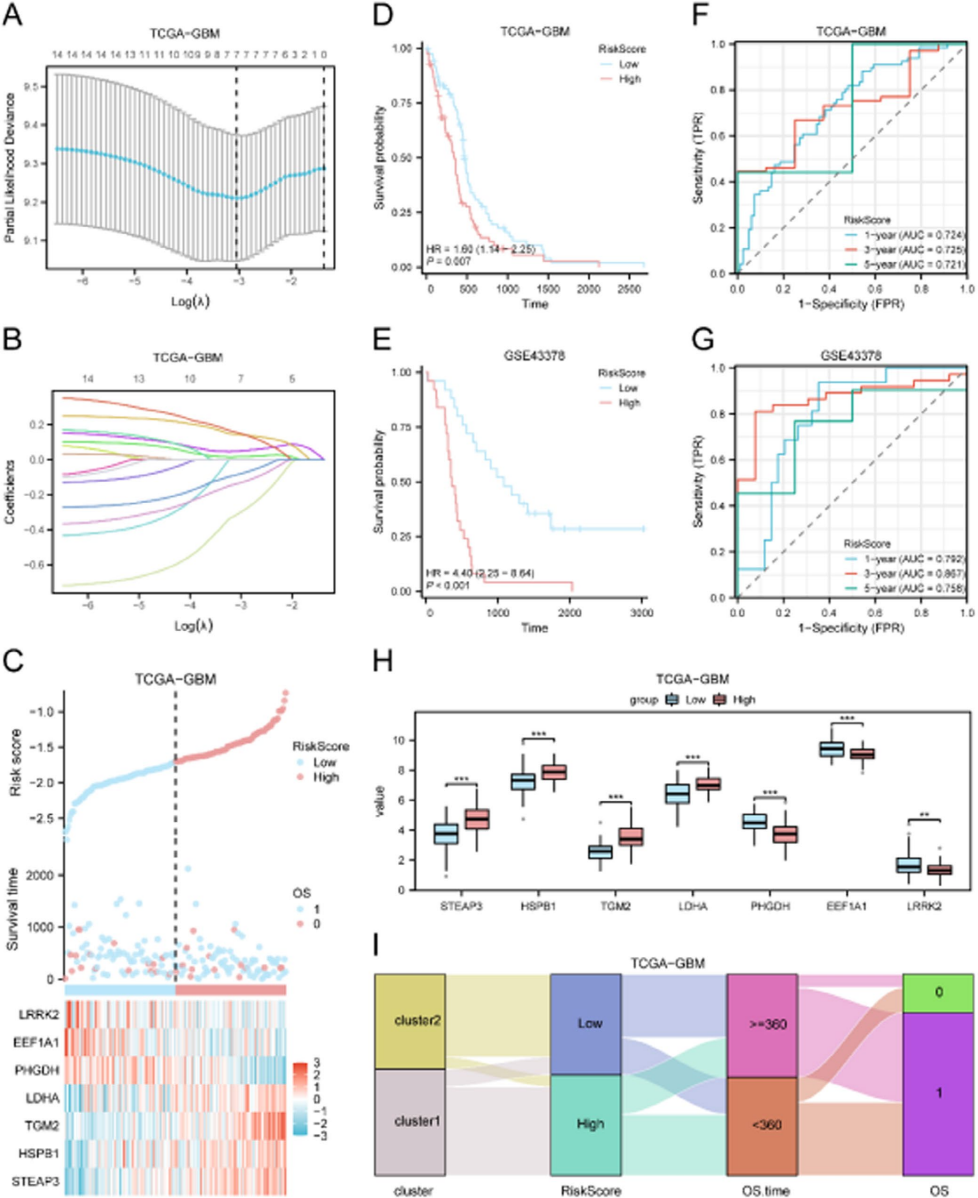
Construction and validation of the prognostic LASSO model. (A) LASSO coefficient profile plot. (B) Cross‐validation for selecting the optimal *λ*. (C) Risk score distribution, survival status, and gene expression heatmap. (D, E) Kaplan–Meier survival curves for high‐ and low‐risk groups in TCGA (D) and GSE43378 (E). (F, G) Time‐dependent ROC curves for 1‐, 3‐, and 5‐year survival prediction in TCGA (F) and GSE43378 (G). (H) Expression differences of 7 key genes between risk groups. (I) Sankey diagram showing associations between subtype, risk group, survival time, and outcome.

LASSO Cox regression identified a 7‐gene prognostic signature (STEAP3, HSPB1, TGM2, LDHA, PHGDH, EEF1A1 and LRRK2) (Figure 12A,B). Patients were stratified into high‐ and low‐risk groups based on the median risk score. The high‐risk group showed significantly worse overall survival in both TCGA and GSE43378 cohorts (Figure 12D,E). Time‐dependent ROC analysis demonstrated robust predictive performance at 1, 3, and 5 years (AUCs: 0.74–0.81; Figure 12F,G).

### 3.10 Regulatory Network Analysis

**FIGURE 13:**
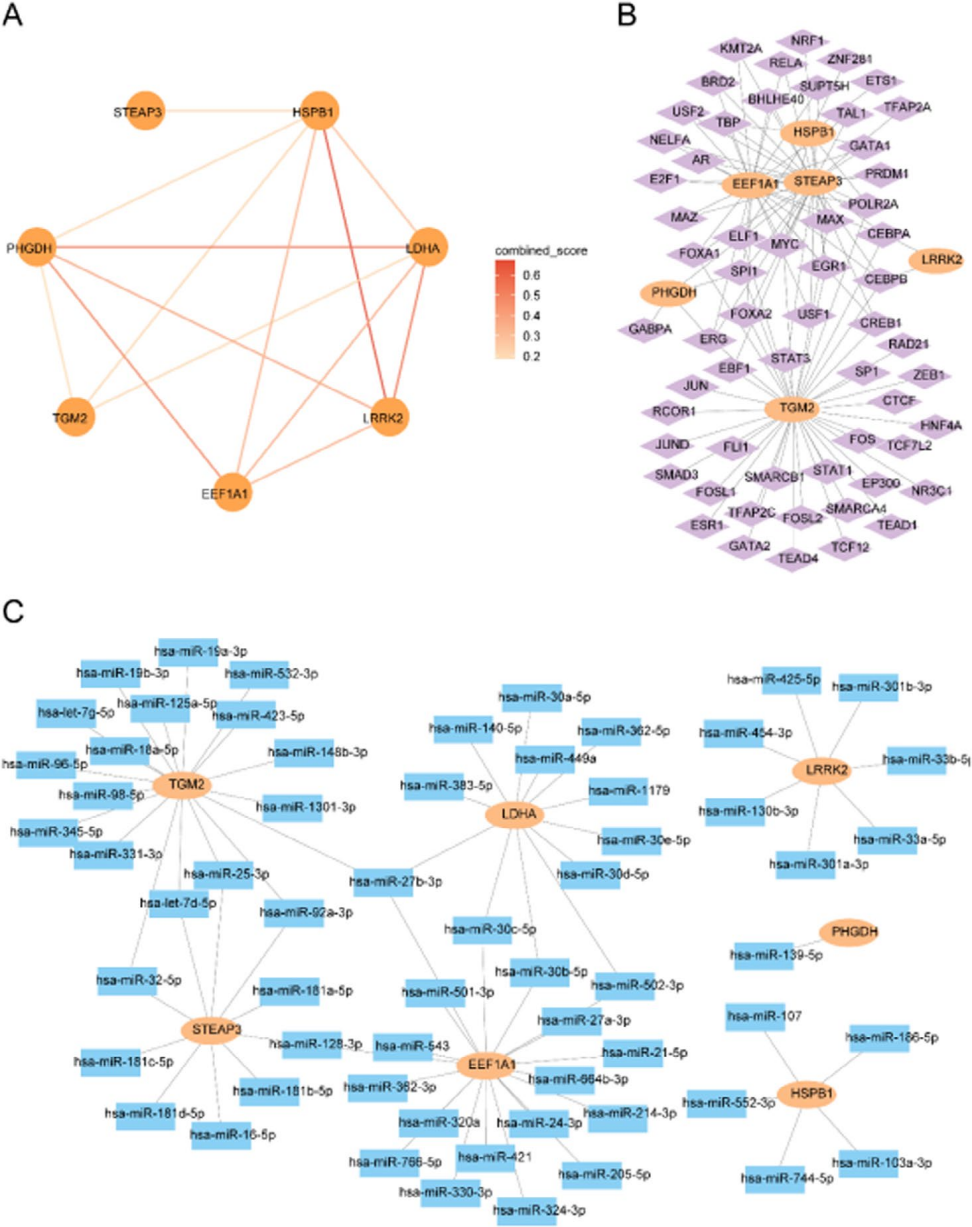
Regulatory networks of key prognostic genes. (A) Protein–protein interaction (PPI) network. (B) mRNA–transcription factor (TF) regulatory network. (C) mRNA–miRNA interaction network. Orange ovals: MRNAs; purple diamonds: TFs; blue squares: MiRNAs.

Protein–protein interaction (PPI) analysis revealed functional connectivity among the seven key genes (Figure 13A). Transcriptional regulatory network analysis identified 59 transcription factors potentially regulating six of the key genes (Figure 13B). Additionally, 62 miRNAs were predicted to target the seven key genes, forming 72 miRNA–mRNA interaction pairs (Figure 13C).

### 3.11 Genomic and Immune Correlates of Risk Groups

**FIGURE 14:**
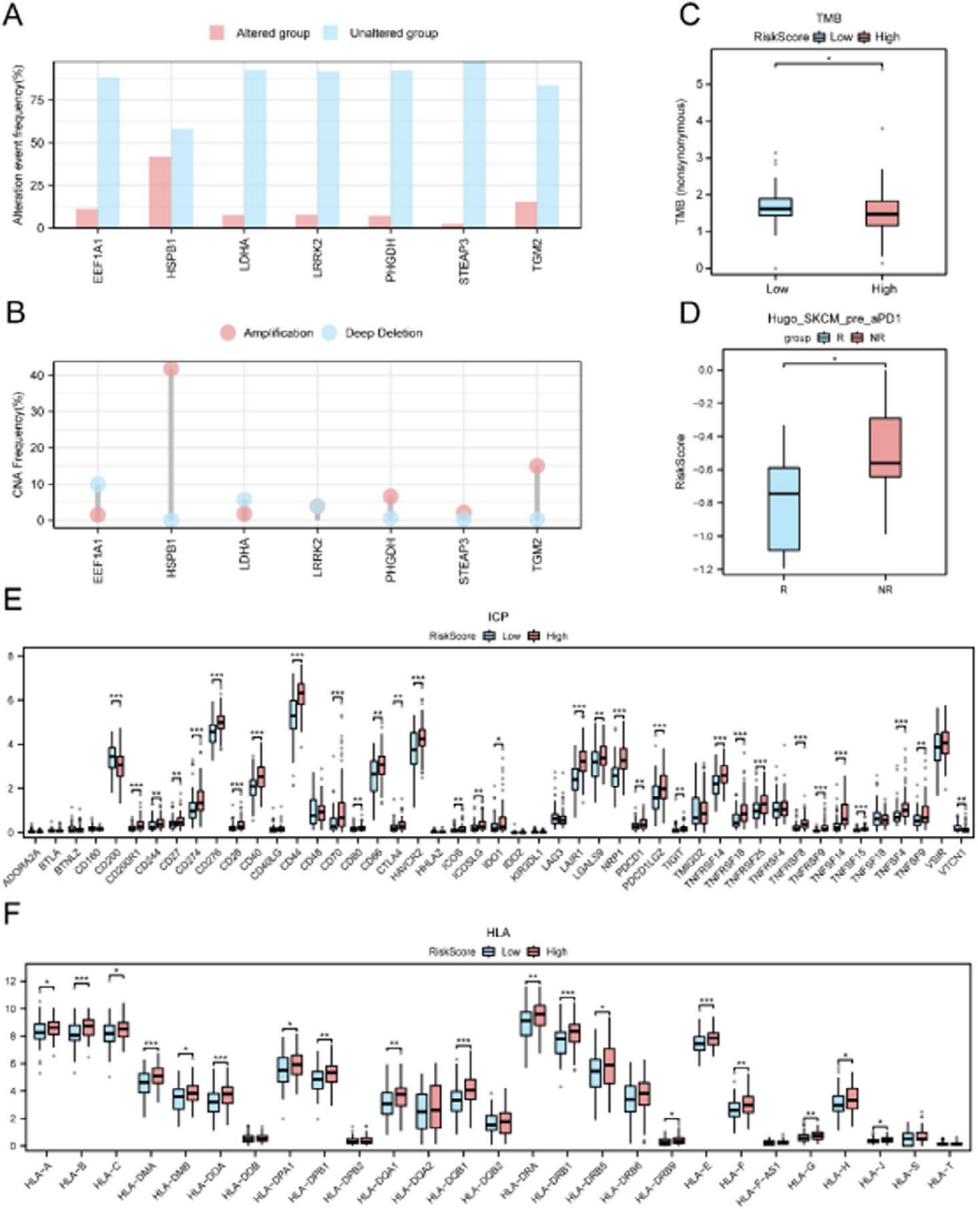
Genomic and immune correlates of risk groups. (A, B) CNV frequency (A) and type distribution (B) for 7 key genes. (C) Tumour mutation burden (TMB) comparison between risk groups. (D) Risk score comparison between responders (R) and non‐responders (NR) in a melanoma immunotherapy cohort. (E, F) Expression differences of immune checkpoint genes (E) and HLA family genes (F) between risk groups.

CNV analysis showed that all seven key genes exhibited copy number alterations, with HSPB1 having the highest frequency (Figure 14A,B). The low‐risk group had significantly higher tumour mutation burden (TMB) compared to the high‐risk group (Figure 14C). In an external melanoma immunotherapy cohort, responders exhibited significantly lower risk scores than non‐responders (Figure 14D). Additionally, 33 immune checkpoint genes and 19 HLA family genes were differentially expressed between risk groups (Figure 14E,F). Regulatory Network and Drug Sensitivity Analysis.

### 3.12 Drug Sensitivity Analysis

**FIGURE 15:**
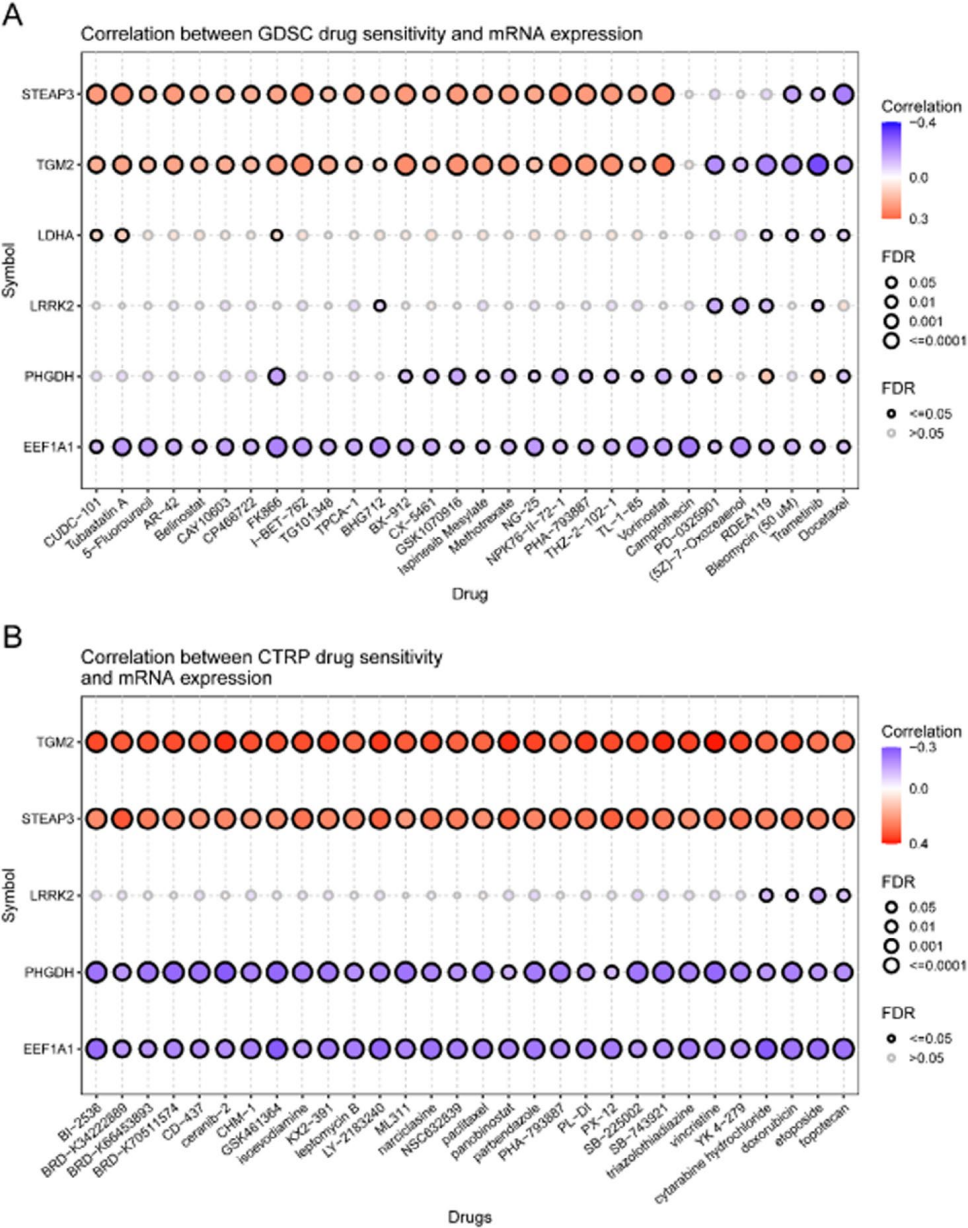
Drug sensitivity analysis. Bubble plots showing correlations between key gene expression and drug sensitivity in GDSC (A) and CTRP (B) databases. Red: Positive correlation; blue: Negative correlation.

Drug sensitivity screening indicated that STEAP3 and TGM2 expression correlated with increased sensitivity to multiple compounds, whereas PHGDH and EEF1A1 were associated with resistance in both GDSC and CTRP databases (Figure 15A,B).

### 3.13 Development of a Clinical Prognostic Nomogram

**FIGURE 16:**
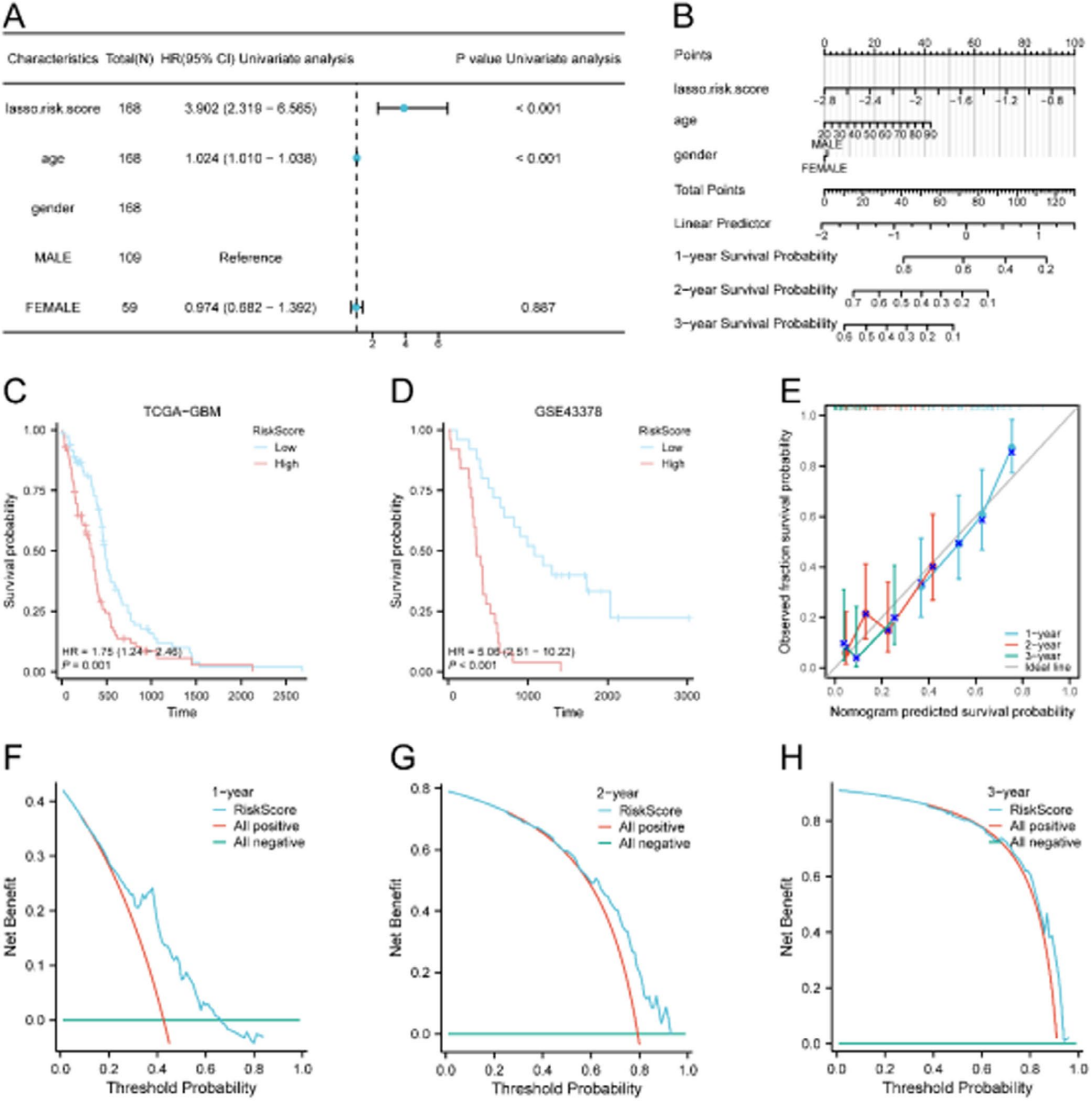
Development and evaluation of a clinical prognostic nomogram. (A) Forest plot of univariate Cox regression results. (B) Nomogram integrating risk score and age for survival prediction. (C, D) Kaplan–Meier survival curves based on nomogram risk score in TCGA (C) and GSE43378 (D). (E) Calibration curves for 1‐, 2‐, and 3‐year survival. (F–H) Decision curve analysis (DCA) for 1‐year (F), 2‐year (G), and 3‐year (H) predictions.

A multivariate Cox model integrating the LASSO risk score and age was developed (Table 5). The nomogram demonstrated good calibration and discriminative ability for predicting 1‐, 2‐, and 3‐year survival (Figure 16B–E). Decision curve analysis (DCA) confirmed its clinical utility across multiple time points (Figure 16F–H).

**TABLE 5:** Cox regression analysis results.

Characteristics	Total (N)	HR (95% CI)	*p*	HR (95% CI)	*p*
		Univariate analysis	Univariate analysis	Multivariate analysis	Multivariate analysis
lasso.risk.score	168	3.902 (2.319–6.565)	< 0.001	3.371 (1.963–5.791)	< 0.001
Age	168	1.024 (1.010–1.038)	< 0.001	1.016 (1.002–1.031)	0.028
Gender	168				
Male	109	Reference			
Female	59	0.974 (0.682–1.392)	0.887		

Abbreviations: GBM, glioblastoma multiforme; OS, overall survival; TCGA, The Cancer Genome Atlas.

## 4 Discussion

We have developed a robust 7‐gene prognostic signature (STEAP3, HSPB1, TGM2, LDHA, PHGDH, EEF1A1 and LRRK2) by integrating the expression profiles of genes involved in mitophagy and oxidative stress regulation. This signature effectively stratified GBM patients into clinically distinct subgroups with significantly different survival outcomes across multiple independent cohorts. Beyond its prognostic value, our analysis reveals that this gene signature delineates a specific tumour phenotype characterized by metabolic reprogramming coupled with an immunosuppressive microenvironment, providing mechanistic insights into GBM progression and therapeutic resistance.

The molecular subtypes (C1 and C2) identified through consensus clustering of prognostic MORGs demonstrated significantly divergent clinical trajectories and biological characteristics. The C2 subtype, correlated with a more favourable prognosis, exhibited enrichment in metabolic pathways such as those involving peroxisomes. In contrast, the aggressive C1 subtype was characterized by the activation of proliferation‐related pathways, including E2F targets and the G2M checkpoint, as well as pro‐inflammatory signalling pathways such as TNFA/NF‐kB and IL6/JAK/STAT3. This observation is consistent with the well‐documented association between cell cycle dysregulation [42] and chronic inflammation [43] and poor prognosis in glioblastoma multiforme (GBM). The pronounced differences in immune cell infiltration and immune checkpoint expression between these subtypes further underscore the potential role of MORGs in shaping an immunosuppressive microenvironment that facilitates tumour progression.

The central focus of our research is the 7‐gene prognostic signature, comprising STEAP3, HSPB1, TGM2, LDHA, PHGDH, EEF1A1 and LRRK2. Each gene within this signature has well‐documented roles in cancer biology, thereby supporting its biological plausibility. LDHA, a critical enzyme in glycolysis, is frequently upregulated in various cancers and facilitates the Warburg effect, which is associated with poor survival outcomes [47]. HSPB1 (also known as HSP27) is known to confer resistance to therapy and is linked to adverse prognoses in gliomas [44]. LRRK2, although primarily recognized for its involvement in Parkinson's disease, has been implicated in the regulation of immune responses and the proliferation of cancer cells [45]. PHGDH, a rate‐limiting enzyme in the serine biosynthesis pathway, is often amplified in cancers and supports anabolic growth [48]. The robust independent prognostic value of this signature, as validated in external datasets, indicates that it encapsulates fundamental aspects of glioblastoma multiforme (GBM) biology, likely integrating elements of metabolic stress, proteostasis and immune evasion.

A crucial discovery is the association between the high‐risk signature and an immunosuppressive tumour microenvironment (TME). Although the high‐risk group demonstrated elevated estimated immune scores, it also exhibited characteristics of immune dysfunction or a ‘cold’ tumour phenotype. This includes increased infiltration of regulatory T cells (Tregs) and macrophages, as well as heightened expression of various inhibitory immune checkpoints, such as CTLA‐4, PD‐1, and TIGIT. This results in a paradoxical situation where the TME is immunologically infiltrated yet functionally suppressed. Such a phenomenon has been observed in other cancers and may elucidate the ineffectiveness of single‐agent immunotherapy in glioblastoma multiforme (GBM) [49, 50]. The positive correlation between the risk score and tumour mutation burden (TMB) is noteworthy, given that high TMB is typically linked to improved responses to immune checkpoint blockade [51]. However, in GBM, which generally exhibits a relatively low TMB, other factors, such as PTEN mutation and myeloid‐driven immunosuppression, may predominate in determining the functional outcome [52, 53]. The markedly lower risk score observed in responders to anti‐PD‐1 therapy in an independent melanoma cohort suggests that the signature may represent a broader immunosuppressive phenotype, potentially valuable for predicting responses to immunotherapy.

The drug sensitivity analysis identified potential therapeutic strategies, demonstrating a positive correlation between STEAP3/TGM2 expression and sensitivity to various pharmacological agents, notably HSP90 inhibitors [46]. This suggests that patients classified as high‐risk could potentially benefit from targeted therapeutic approaches. In contrast, the observed negative correlation for PHGDH and EEF1A1 indicates possible resistance mechanisms. These in silico predictions offer a basis for drug repurposing and the development of combination therapy strategies tailored to specific risk subgroups, although experimental validation is necessary to confirm these findings.

Several limitations of this study merit acknowledgment. First, the retrospective nature of the public data utilized introduces potential biases, necessitating prospective validation within a dedicated clinical cohort, ideally one that is prospective in design. Second, the biological mechanisms by which these seven genes interact to influence the glioblastoma multiforme (GBM) tumour microenvironment (TME) and prognosis remain largely unexplored [54]. Functional studies employing GBM cell lines and animal models are imperative to establish causality and elucidate the underlying mechanisms [55]. Third, although validation datasets were included, the sample size for the analysis of immunotherapy response was limited. Larger cohorts with matched clinical response data to immunotherapy are required to substantiate the predictive value of the signature.

In conclusion, we have developed and validated a novel 7‐gene signature that integrates mitophagy and oxidative stress pathways to predict GBM prognosis. This signature identifies a high‐risk subgroup characterized by metabolic reprogramming and immunosuppression, providing insights into the biological basis of therapeutic resistance. Our findings highlight the interconnectedness of cellular metabolism, mitochondrial homeostasis, and antitumor immunity in GBM progression. Further validation and mechanistic studies of this signature could facilitate the development of personalized treatment strategies targeting these vulnerabilities in aggressive GBM.

## Author Contributions

**Yang Wang:** conceptualization, software, investigation, validation, formal analysis. **Hongliang Zhong:** supervision, visualization, investigation, formal analysis. **Changjiang He:** writing – review and editing, writing – original draft, project administration, data curation, methodology, resources.

## Funding

This study was not funded by anything.

## Ethics Statement

This study utilized publicly available datasets from The Cancer Genome Atlas (TCGA) and the Gene Expression Omnibus (GEO). All data were de‐identified. The original studies obtained ethical approval and patient consent. Therefore, this secondary analysis did not require additional ethical approval from our institutional review board.

## Consent

The authors have nothing to report.

## Conflicts of Interest

The authors declare no conflicts of interest.

## Data Availability

The original datasets analysed in this study are publicly available. The transcriptomic and clinical data for the TCGA‐GBM cohort can be accessed via the Genomic Data Commons (GDC) portal (https://portal.gdc.cancer.gov/) under the project ‘TCGA‐GBM’. The validation datasets GSE43378 and GSE147352 are available from the Gene Expression Omnibus (GEO) database (https://www.ncbi.nlm.nih.gov/geo/) under the accession numbers GSE43378 and GSE147352, respectively. The mitophagy and oxidative stress‐related gene (MORG) list, the derived 7‐gene signature, and the analysis codes supporting the findings of this study are available from the corresponding author upon reasonable request.
